# Severe Paradoxical Manifestations in an Immunocompetent Young Female With Tuberculous Meningitis

**DOI:** 10.7759/cureus.29953

**Published:** 2022-10-05

**Authors:** Sangeeta Gupta, Upasna Sinha, Amit Raj

**Affiliations:** 1 Physiology, All India Institute of Medical Sciences, Gorakhpur, Gorakhpur, IND; 2 Radiology, All India Institute of Medical Sciences, Patna, Patna, IND; 3 Ophthalmology, All India Institute of Medical Sciences, Patna, Patna, IND

**Keywords:** magnetic resonance imaging, paradoxical manifestation, human immuno-deficiency virus, tuberculous meningitis, anti-tuberculosis treatment

## Abstract

A paradoxical reaction, in patients with tuberculous meningitis, is described as either worsening of pre-existing tuberculous lesions or the appearance of new tuberculous lesions after initial improvement with anti-tuberculosis treatment. The condition is often difficult to predict. When severe, it may also result in considerable neurological morbidity and even death. We aim to report such a rare case of severe paradoxical response in a young female with tuberculous meningitis. An 18-year-old female developed severe headache, vomiting, altered sensorium, decreased vision, and paraparesis. She was on anti-tuberculosis treatment (ATT) for tuberculous meningitis for the past two months. Radiological findings revealed tuberculomas in the left cerebral and cerebellar hemispheres, adhesive arachnoiditis in the cervical spine, and moderate communicating hydrocephalus. Visual evoked potential tests provided electrophysiological evidence of optic neuropathy in the left eye. The appearance of new (tuberculoma and adhesive arachnoiditis in the cervical spine), as well as aggravation of pre-existing tuberculous lesions (worsened hydrocephalus and worsened clinical features), were evident in the patient, suggestive of severe paradoxical response (with HIV negative serology). The patient was treated with corticosteroids, and antibiotics, and continued the ATT programme in a conservative manner. Nonetheless, as the patient had severe CNS manifestations, severe disabilities (poor vision, paraparesis, or quadriparesis) and fatalities were inevitable. Notwithstanding, it is crucial to recognize the paradoxical manifestations of tuberculous meningitis to avoid misleading diagnoses and unwarranted management strategies.

## Introduction

Tuberculous meningitis (TBM) is the most dreaded complication of tuberculosis owing to its high mortality and morbidity rates [1]. Tuberculosis has reached epidemic proportions in both developed and underdeveloped nations. CNS involvement, however, has been reported to be more in developing countries with about 10% of extrapulmonary tuberculosis resulting in tuberculous meningitis [2, 3]. There has also been a greater association of paradoxical response with this form of tuberculosis. Moreover, some severe forms of paradoxical manifestations reported in this condition (like optochiasmatic and spinal arachnoiditis resulting in vision loss and paraplegia) are inherently devastating. Paradoxical reactions in tuberculous meningitis can be erroneously concluded either as treatment failure, drug resistance, drug toxicity, presence of malignancy, or clinical deterioration due to other infections or conditions. Hence, recognizing and distinguishing them from the aforementioned conditions becomes crucial. Clinical and radiological deterioration associated with such reactions can mislead and predispose one to look for an alternative diagnosis.

A paradoxical reaction, in tuberculous meningitis, is characterised by either worsening of pre-existing tuberculous lesions or the presence of new tuberculous lesions in patients with initial improvement and on anti-tuberculosis treatment for at least 10 days [4-7]. Concomitant HIV infection, female gender, and a shorter duration of illness have been reported as important predictors of paradoxical reactions in patients with tuberculous meningitis [8].

Paradoxical reactions have been reported in varying proportions in HIV-negative patients with TB, with as low as <1%. The mean duration of paradoxical reaction has been suggested as 82.45 days (16-320 days) [8].

Paradoxical manifestations are described as the appearance of the clinical features and/or neuroimaging abnormalities or their aggravation during anti-tuberculosis treatment. Clinical features include fever, headache, vomiting, altered sensorium, decreased vision, seizures, vision loss, cranial nerve palsies, raised intracranial pressure and focal neurological deficits [9]. Neuroimaging abnormalities include hydrocephalus, infarction, basal exudates, tuberculoma and angiographic abnormalities [10]. While the majority of the patients with paradoxical reactions have been reported to have favourable outcomes with appropriate diagnosis, some rare severe forms are known, which are dreadful, resulting in catastrophic outcomes with increased mortality and morbidity. We present a case of tuberculous meningitis in an HIV-negative young female who developed grave forms of paradoxical reactions resulting in a severe diminution of vision and altered sensorium after about eight weeks of antituberculosis therapy. The report aims to emphasize this rare situation and increase clinicians' awareness.

## Case presentation

**Figure 1:**
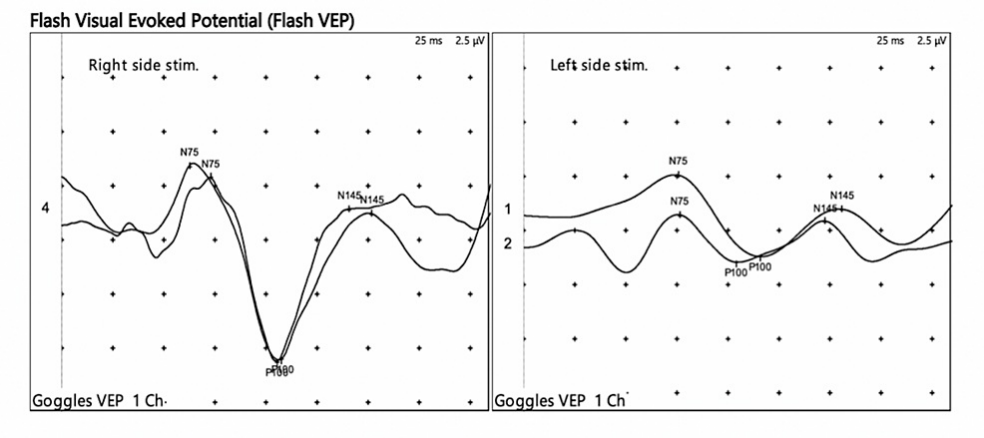
Flash VEP record in the left eye reveals poor reproducibility and severely reduced inter-ocular amplitude ratio (>80% reduction). The right eye flash VEP record exhibits no abnormal findings. VEP: visual evoked potential

An 18-year-old female presented with an altered level of consciousness (Glasgow coma scale of 3: E1V1M1) preceded by fever, headache and vomiting. Her pulse rate was 138 beats/min while her blood pressure was 98/66 mm Hg. The temperature was 101 °F. Her pupils were mid-dilated bilaterally and sluggishly reacting to light. She was under antituberculosis treatment (ATT) for about one and a half months as a combination of rifampicin (10 mg/kg/day), isoniazid (5 mg/kg/day), pyrazinamide (25 mg/kg/day) and ethambutol (15 mg/kg/day) for tuberculous meningitis. Her previous MRI brain scan revealed the signs of meningitis and the presence of mild hydrocephalus with no other lesions. Her CSF (cerebrospinal fluid) examination revealed a protein level of 272.8 mg/dl and a glucose level of 26.9 mg/dl. The total number of cells was 442/cubic mm (82% were lymphocytes). A diagnosis of tuberculous meningitis was made, based on the clinical, CSF examination and the radiological findings (meningeal enhancement and mild hydrocephalus). Intravenous dexamethasone (0.4 mg/kg/day) and symptomatic treatment (antipyretics) were started. Antituberculosis treatment (ATT) (rifampicin, isoniazid, pyrazinamide and streptomycin in recommended dosages) was continued. She was in full compliance with the antituberculous drug regime with gradual improvement in her clinical condition (Glasgow coma scale: E2V7M5, afebrile and no complaints of vomiting) after about four days. However, after being compliant with the ATT therapy as prescribed, she presented with severe headache and vomiting along with a severe diminution of vision and difficulty in walking (after 15 days of hospital admission and two months of commencement of ATT). On examination, fundoscopy (Heine beta 200 LED ophthalmoscope) showed bilateral optic atrophy. Visual field examination (perimetry) revealed a visual loss in bilateral temporal fields. Visual evoked potential tests were performed which depicted reduced inter-ocular amplitude ratios (relative reduction of amplitudes in the left eye) and poorly replicable waveforms in the left eye. The test provided electrophysiological evidence of optic neuropathy in the left eye (reduced amplitude) (Figure 1).

**Figure 2:**
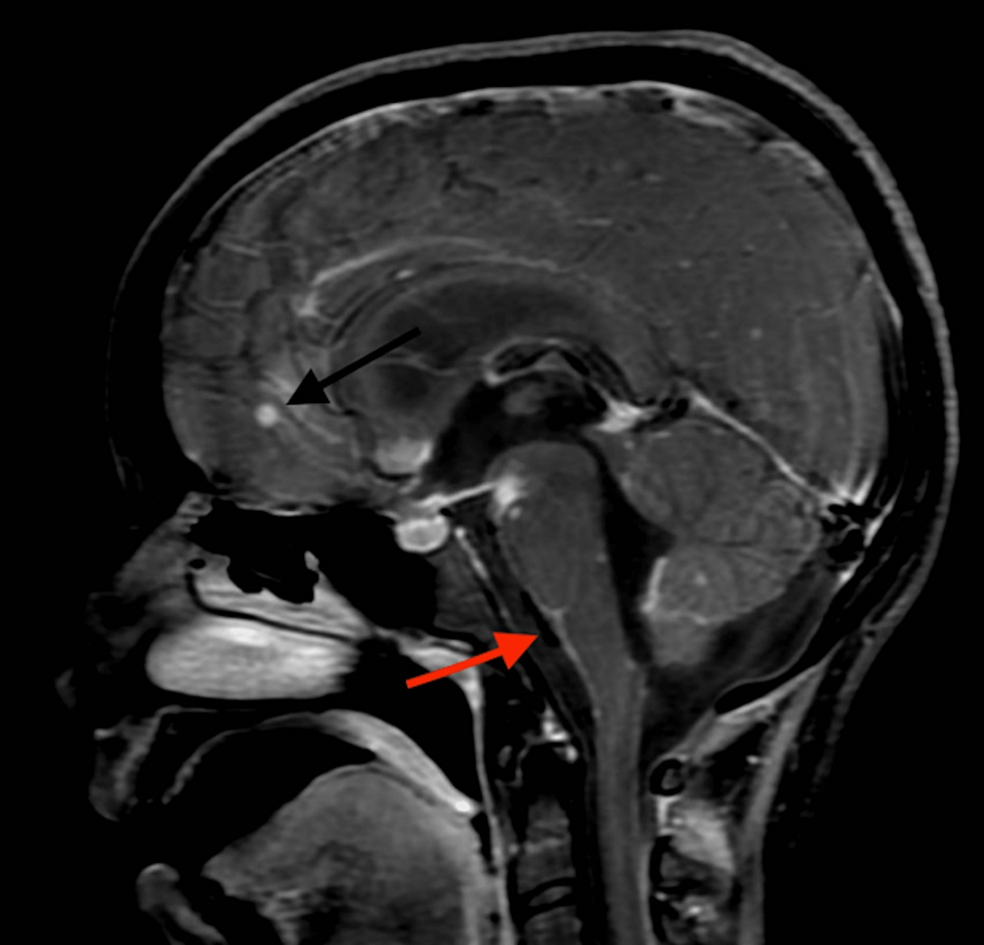
Contrast-enhanced T1 weighted sagittal image showing nodular enhancing lesions (black arrow) with meningeal enhancement along the surface of the brainstem (red arrow)

**Figure 3:**
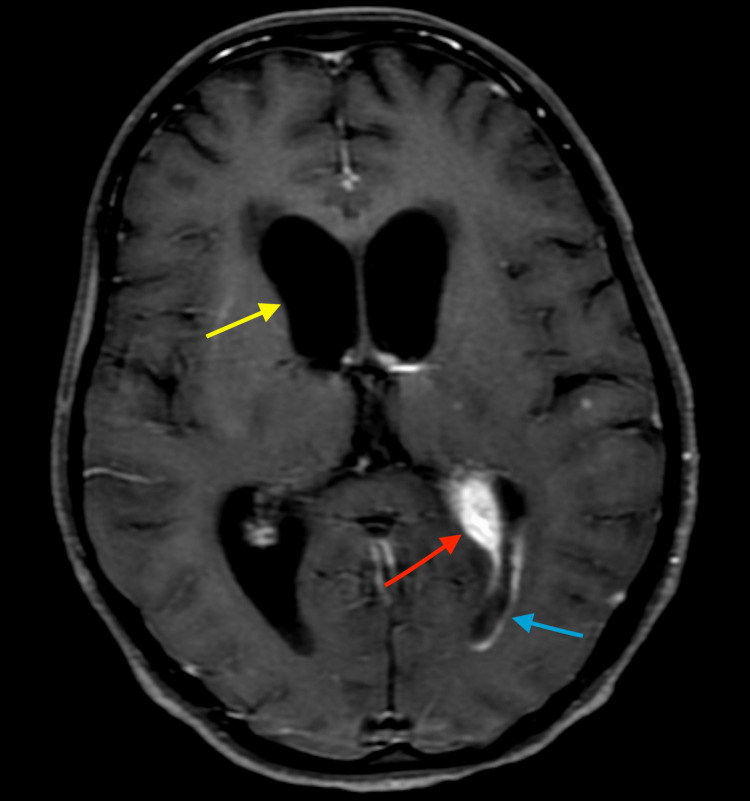
Contrast-enhanced T1 weighted axial image shows hydrocephalus (yellow arrow) with enlarged and enhancing left choroid plexus (choroid plexitis) (red arrow) and enhancement along the wall of the occipital horn of the left lateral ventricle (ventriculitis) (blue arrow).

Her serology (ELISA: enzyme-linked immunosorbent assay) was negative for HIV. According to the CSF (cerebrospinal fluid) culture, mycobacterium tuberculosis was found to be sensitive (bacterial culture and susceptibility test) to the anti-tuberculous drugs being administered to the patient. The brain MRI (plain and contrast) was performed on a 3T scanner with 32 channel coil for minIP, magnitude and phase images and contrast T1 weighted-axial, coronal and sagittal images. Brain MRI revealed a few tiny nodular enhancing areas in the region of left frontal, temporal, parietal and occipital regions and left cerebellar hemispheres suggestive of tuberculoma (Figure 2). Features of meningitis with communicating hydrocephalus of moderate degree were evident. Features of choroid plexitis and ventriculitis in the left antrum and left occipital horn were also found (Figure 3). Also, an area of altered signal intensity in the region of left basal ganglia was found which may be one of the secondary ischaemic changes. Screening of cervical spine shows adhesive arachnoiditis with CSF trapping. There was a posterior displacement of the spinal cord up to the C7 vertebra and anterior displacement of the spinal cord at the D1-D5 level.)

Clinical features and radio-imaging findings supported the presence of tuberculomas in the left cerebral and cerebellar hemispheres along with adhesive arachnoiditis in the cervical spine and moderate communicating hydrocephalus. The findings are concordant with her neuro-ophthalmologic manifestations and are suggested to be due to the paradoxical response to the treatment. The appearance of new tuberculous lesions after having improved clinical symptoms initially and continued antituberculosis treatment for the last two months were suggestive of the development of paradoxical reactions. The patient was continued on ATT. Her urine culture was positive for Klebsiella pneumoniae (asymptomatic bacteriuria) and sensitive to amikacin. Amikacin (500 mg BD) was started. Meropenem was also given as a broad-spectrum antibiotic (2 gm stat followed by 1 gm TDS). A ventro-peritoneal shunt was planned but could not be performed as consent was not given by the patient’s relatives. Systemic corticosteroid administration was followed by oral prednisolone (20 mg OD) and the patient was managed conservatively along with physiotherapy for lower limb paresis. Owing to the improvement in her clinical condition (with GCS 15 and normal vitals), she was discharged after about 15 days with a continued ATT regime (isoniazid, rifampicin, pyrazinamide and streptomycin) and continued steroid treatment (oral prednisolone with gradual tapering doses). The poor prognosis of vision was explained to her. The patient died after about 10 days of discharge (after about 25 days of development of paradoxical manifestations).

## Discussion

Appearance or aggravation of clinical features and/or neuroimaging abnormalities during anti-tuberculosis treatment (after at least 10 days of anti-tuberculosis treatment) have been described as paradoxical manifestations in tuberculous meningitis. The afore-mentioned case was on anti-tuberculosis treatment for about two months when she developed severe headache, vomiting, decreased vision and paraparesis along with the presence of tuberculomas in the left cerebral and cerebellar hemispheres along with adhesive arachnoiditis in the cervical spine (new lesions) and moderate communicating hydrocephalus (worsening) in the radiological examination. Hence, the appearance of new tuberculous lesions as well as aggravation/worsening of pre-existing tuberculous lesions were remarkable in the patient. As the patient deteriorated after about two months of commencement of the treatment, the paradoxical manifestations in this patient represent the "definite" category according to Carvalho et al. [11] The time of onset and the clinical/radiological worsening of previous tuberculous lesions along with the development of new lesions mitigated the possibility of the presence of delayed treatment response.

Concomitant HIV infection, which is reported to be the significant predictor of paradoxical reactions, was not a feature in our patient. A paradoxical response is particularly seen in adult and immunocompromised patients. This rate is lower than 5% in TB patients with normal immune systems [12]. Also, some risk factors have been found to be associated with paradoxical reactions like anaemia, hypoalbuminemia, and lymphopenia [13]. But, there were none of these aforementioned risk factors in our patient. A paradoxical response can occur 3-12 weeks after the initiation of a TB treatment; however, it can take as long as 18 months [14]. It was seen about two months after the initiation of the treatment in the present case.

Paradoxical deterioration has more often been associated with extrapulmonary and disseminated TB, like miliary tuberculosis and tuberculous meningitis. CNS manifestations in the patients often include headache, mental confusion, focal seizures, cranial nerve palsies, hemiparesis, paraparesis, and hemianaesthesia as a result of the enlargement or development of intracranial tuberculomas and hydrocephalus [15]. Our patient presented with similar CNS manifestations of severe headache, mental confusion and paraparesis caused by enlargement of the CNS lesions. 

The cause of the development of a paradoxical response is not clearly known. Various hypotheses have been suggested to explain this phenomenon. An excessive inflammatory response against mycobacterial antigens has been the suggested underlying cause. Mycobacterial cell wall antigens present in the affected brain tissues, trigger an exaggerated inflammatory reaction following treatment with anti-tuberculosis drugs.

More robust immune responses to antigenic challenges, like infection and vaccination are reported to be exhibited in females as compared to males. The presence of specific receptors for sex hormones on immune cells and their responses to changes in hormone levels can result in the modulatory effect of female sex hormones on the immune response towards Mycobacterium tuberculosis-associated antigens. This might explain the gender variation associated with the paradoxical response [16,17].

Optochiasmatic and spinal arachnoiditis and a large cerebral tuberculoma are serious conditions reported to be associated with severe disabilities, and potential for death [18]. These conditions warrant urgent treatment with immunomodulatory drugs. High doses of corticosteroids are currently the most preferred treatment. The death of the patient in the present study can be a consequence of the development of the above-mentioned serious CNS manifestations. These devastating complications of tuberculous meningitis often occur as a result of increased intracranial pressure and/or compression over the visual pathways. Ventro-peritoneal shunt in the patient could have contributed to ameliorating the condition which was unfortunately disallowed by the patient’s relatives.

## Conclusions

A paradoxical response may lead to perplexity in the management of TBM disease. Understanding the nature and recognizing the paradoxical response can help to avoid unnecessary management strategies. Although paradoxical responses have not been associated with adverse outcomes in several previous studies, it has been stated substantially that manifestations of paradoxical reactions like optochiasmatic and spinal arachnoiditis, as well as a large cerebral tuberculoma, are serious conditions associated with severe disabilities and are also potential causes of death. It is clear that recognizing the condition and the right diagnosis as well as excluding other misleading conditions like multidrug resistance, the possibility of treatment failure, drug toxicity, and clinical deterioration are crucial and decisive in the optimal management of the condition.
